# CNS SIRT3 Expression Is Altered by Reactive Oxygen Species and in Alzheimer’s Disease

**DOI:** 10.1371/journal.pone.0048225

**Published:** 2012-11-06

**Authors:** Heather J. M. Weir, Tracey K. Murray, Patrick G. Kehoe, Seth Love, Eric M. Verdin, Michael J. O’Neill, Jon D. Lane, Nina Balthasar

**Affiliations:** 1 School of Biochemistry, University of Bristol, Bristol, United Kingdom; 2 Neurodegenerative Diseases Drug Hunting Team, Eli Lilly and Co. Ltd., Windlesham, Surrey, United Kingdom; 3 Dementia Research Group, Institute of Clinical Neurosciences, University of Bristol, Bristol, United Kingdom; 4 Gladstone Institute of Virology and Immunology, University of California San Francisco, San Francisco, California, United States of America; 5 School of Physiology and Pharmacology, University of Bristol, Bristol, United Kingdom; Virginia Commonwealth University, United States of America

## Abstract

Progressive mitochondrial dysfunction contributes to neuronal degeneration in age-mediated disease. An essential regulator of mitochondrial function is the deacetylase, sirtuin 3 (SIRT3). Here we investigate a role for CNS Sirt3 in mitochondrial responses to reactive oxygen species (ROS)- and Alzheimer’s disease (AD)-mediated stress. Pharmacological augmentation of mitochondrial ROS increases *Sirt3* expression in primary hippocampal culture with SIRT3 over-expression being neuroprotective. Furthermore, *Sirt3* expression mirrors spatiotemporal deposition of β-amyloid in an AD mouse model and is also upregulated in AD patient temporal neocortex. Thus, our data suggest a role for SIRT3 in mechanisms sensing and tackling ROS- and AD-mediated mitochondrial stress.

## Introduction

Decline in cognition is closely associated with age-related structural and functional changes of neurons leading to synaptic dysfunction [1]. Synaptic activity is critically dependent on robust mitochondrial function for sufficient supply of ATP. Pathological ageing involving the accumulation of β-amyloid (Aβ, a cleavage product of Amyloid Precursor Protein (APP)) in Alzheimer’s disease (AD) further illustrates the involvement of mitochondrial dysfunction in neurodegenerative disease: mutant APP and Aβ enter mitochondria and interact with mitochondrial proteins, thereby disrupting the electron transport chain (ETC), increasing reactive oxygen species (ROS) to damaging levels and inhibiting the generation of ATP [2], [3].

A mitochondrial protein that has been shown to be critical for the maintenance of appropriate ROS levels and ATP output is the sirtuin family member, SIRT3 [4], [5]. SIRT3 has, after some controversy, now convincingly been demonstrated to reside in the mitochondrial matrix and is expressed in multiple tissues including kidney, heart, liver, adipose tissue and brain [6]. SIRT3 is upregulated in response to fasting and calorie restriction and has been shown to reduce ROS levels in adipocytes [7] and cardiomyocytes [8] and to reduce oxidative damage and enhance the mitochondrial glutathione antioxidant defense system in cochlear neurons [9]. Substrates activated by SIRT3-mediated deacetylation include a number of proteins critical for the maintenance of mitochondrial metabolic balance[4], [5], [9]–[15], and SIRT3-deficient mice show significantly enhanced acetylation of these, leading to increased oxidative damage in multiple tissues [9], [16]. Furthermore, Kim *et al.* demonstrated that SIRT3 is neuroprotective against NMDA-mediated excitotoxicity *in vitro* [17].

Given its role in maintaining mitochondrial function, we hypothesized that SIRT3 might be involved in the mitochondrial mechanisms sensing and tackling conditions of pathological neuronal dysfunction, such as during oxidative stress in AD.

## Results

### Mouse CNS SIRT3 is Expressed in Neurons and Glia with Long-form SIRT3 being Exclusively Mitochondrial

**Figure 1:**
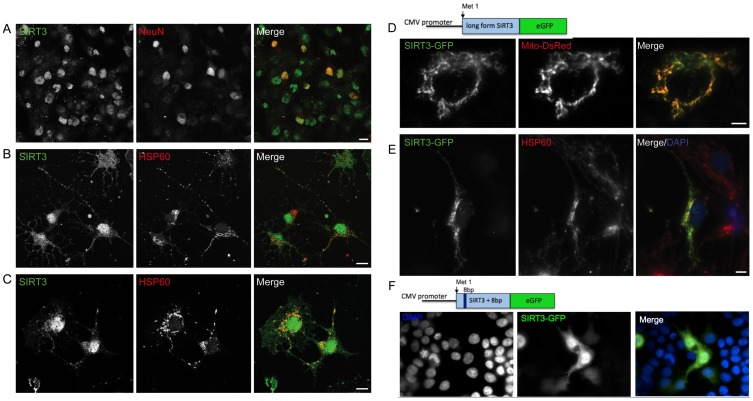
Subcellular localization of CNS SIRT3. Confocal analysis of SIRT3 subcellular localization: **A.** SIRT3 is expressed in neurons and glia. *Left*: SIRT3 immunohistochemistry in primary hippocampal cultures. *Middle:* NeuN, *Right*: red and green channels merged. **B, C.** SIRT3 expression localizes to the nucleus and mitochondria of the cell body, axons and dendrites. *Left*: SIRT3 immunohistochemistry. *Middle:* mitochondrial HSP60, *Right*: red and green channels merged. Scale bar A–C = 10 µm **D and E.** Over-expression of long-form SIRT3eGFP results in exclusively mitochondrial localization. D. HeLa cells were co-transfected with Mito-DsRed and ‘long-form’ SIRT3eGFP plasmids (left: green SIRT3eGFP, middle: red Mito-DsRed, right: merge of channels). E. Primary hippocampal cultures were transfected with a ‘long-form’ SIRT3eGFP plasmid, fixed and immunohistochemistry performed for HSP60 (middle: red) and eGFP (left: green, right: merge of channels). Scale bar D, E = 5 µm. **F.** Over-expression of ‘short-form’ Sirt3eGFP results in cytoplasmic and nuclear localization. HEK293T cells were transfected with ‘short-form’ SIRT3eGFP, fixed and labeled with DAPI (left panel) and anti-GFP (middle panel, right: merge of channels).

Initially we investigated SIRT3 protein expression in the mouse CNS by immunohistochemistry. SIRT3 was expressed throughout the CNS, although not in every cell, with labeling appearing strongest in nuclei (Fig. S1). For further analysis of CNS SIRT3 subcellular localization, we generated hippocampal primary cultures; co-staining of SIRT3 with NeuN revealed that SIRT3 was expressed in neurons as well as glia (Fig. 1A). Co-localization of SIRT3 with the mitochondrial protein HSP60 demonstrated that SIRT3 was present in somatic, as well as dendritic and axonal mitochondria (Fig. 1B, 1C). Labeling for SIRT3 was also detected in the nucleus.

In mouse, two main splice variants of *Sirt3* generate either ‘long-form’ SIRT3 containing an N-terminal mitochondrial localization signal (MLS) or ‘short-form’ SIRT3 starting at a methionine 78 amino acids downstream [18]. The SIRT3 antibody used for immunohistochemistry recognizes an epitope at the C-terminus of SIRT3; images shown in Fig. 1A–C are thus likely to reflect the localization of both long- and short-forms of SIRT3. We cloned both forms of *Sirt3* from mouse brain cDNA and to analyze localization of both forms of SIRT3 we generated ‘long-form’ and ‘short-form’ *Sirt3* C-terminal-eGFP fusion constructs. Transfection of ‘long-form’ SIRT3eGFP into HeLa cells and primary hippocampal cultures resulted in exclusively mitochondrial staining for SIRT3, as indicated by co-localization with mitochondrial-targeted dsRed (Fig. 1D) or with the anti-HSP60 antibody (Fig. 1E) respectively. In contrast, transfection of ‘short-form’ SIRT3eGFP into HEK293T cells resulted in cytoplasmic and nuclear SIRT3 localization, with no apparent mitochondrial SIRT3eGFP (Fig. 1F).

### In vitro Pharmacological Interference with the Mitochondrial ETC Upregulates Sirt3 mRNA Expression

**Figure 2:**
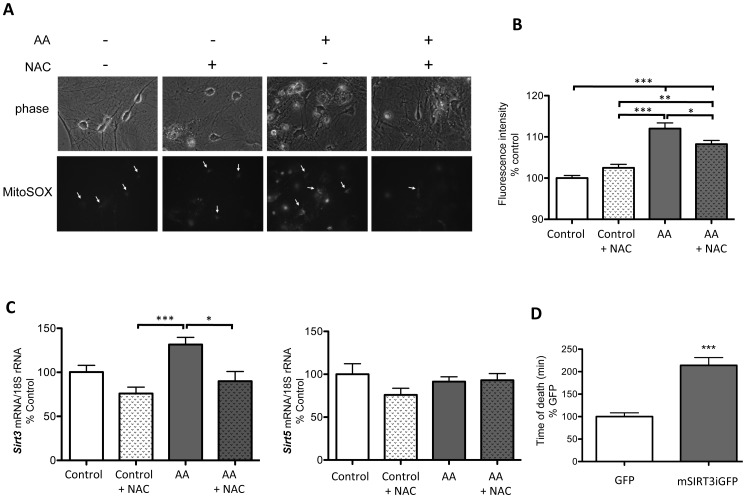
CNS Sirt3 mRNA expression is regulated by mitochondrial ROS and Sirt3 over-expression increases neuronal longevity. **A.** Primary hippocampal cultures were loaded with MitoSOX, treated with antimycin A (AA, 250 nM, 12 hr) and/or pre-treated with N-acetyl-L-cysteine (NAC, 100 µM, o/n) and phase and fluorescent still images taken at 12 hrs. **B.** Mitochondrial ROS is significantly increased following AA treatment, which is partially blunted by NAC (n ≥50 neurons per treatment, one-way ANOVA ***P<0.001, **P<0.01, *P<0.05). **C.** *Sirt3*, but not *Sirt5* mRNA expression is upregulated in response to AA treatment in primary hippocampal cultures, which is blocked by NAC ROS scavenging (n = 10). *Sirt3/5* mRNA was measured and normalized to 18S rRNA using TaqMan multiplex QPCR. **D.** Sirt3 over-expression significantly increases neuronal lifespan. Hippocampal primary cultures were transduced with neuronal-specific lenti-GFP (control) or lenti-mSIRT3iGFP lentivirus and treated with AA (250 nM). Time until fluorescent neuronal death was recorded and is expressed as %control (n>150, ***P>0.0001).

Since mitochondrial oxidative stress is a hallmark of several neuropathological diseases, including AD, we aimed to investigate whether interference with the ETC and induction of ROS could trigger changes in *Sirt3* expression. Treatment of primary hippocampal cultures with antimycin A (AA, an ETC complex III inhibitor [19]) did indeed increase mitochondrial ROS levels in primary hippocampal neurons, as demonstrated by significant increases in MitoSOX fluorescence (Fig. 2A, 2B). Addition of the antioxidant, ROS-scavenging N-acetyl-L-cysteine (NAC), significantly reduced MitoSOX fluorescence and thus mitochondrial ROS levels (Fig. 2A, 2B). AA treatment caused an increase in *Sirt3* mRNA expression, whilst NAC-mediated reduction of mitochondrial ROS completely blocked AA’s effect on *Sirt3* mRNA expression (Fig. 2C). Mitochondrial oxidative stress due to AA treatment had no effect on mitochondrial *Sirt5* mRNA expression (Fig. 2C). To assess whether *Sirt3* splicing might be involved in the cells’ response to mitochondrial oxidative stress, we measured long- or short-form-specific regulation of *Sirt3* mRNA in response to AA treatment. We designed long- and short-form-specific mouse Sirt3 TaqMan qRT-PCR assays and confirmed their specificity in rat PC12 cells transfected with either long-or short-form mouse Sirt3eGFP. Note that mouse Sirt3 TaqMan probes do not recognize rat Sirt3 (Fig. S2A–C). We went on to measure AA (and thus ROS)-mediated *Sirt3* mRNA regulation in primary hippocampal cultures and confirmed significant up-regulation of ‘total’ *Sirt3* mRNA (Fig. S2D). Using the long- and short-form-specific Sirt3 probes we found that both splice variants were up-regulated in response to oxidative stress (Fig. S2E, S2F) and determined that indeed the ratio of long- to short-form *Sirt3* mRNA does not change (Fig. S2G).

### Lentiviral Long-form Sirt3 Over-expression Increases Neuronal Longevity

Given *Sirt3*’s regulation by mitochondrial increases in ROS, we examined whether increases in *Sirt3* may be part of a neuroprotective response to mitochondrial stress. Primary hippocampal cultures were transduced with lentivirus expressing either GFP (lenti-GFP) or long-form mouse Sirt3 cDNA coupled to an IRES-GFP (lenti-mSIRT3iGFP), both driven by a neuronal-specific synapsin promoter (see Fig. S3A–C) and treated with AA to increase mitochondrial ROS. Sirt3 over-expression significantly increased neuronal life span of fluorescent neurons (Fig. 2D and S3D, S3E).

### Sirt3 Expression is Upregulated in a Mouse Model Over-expressing Aβ

**Figure 3:**
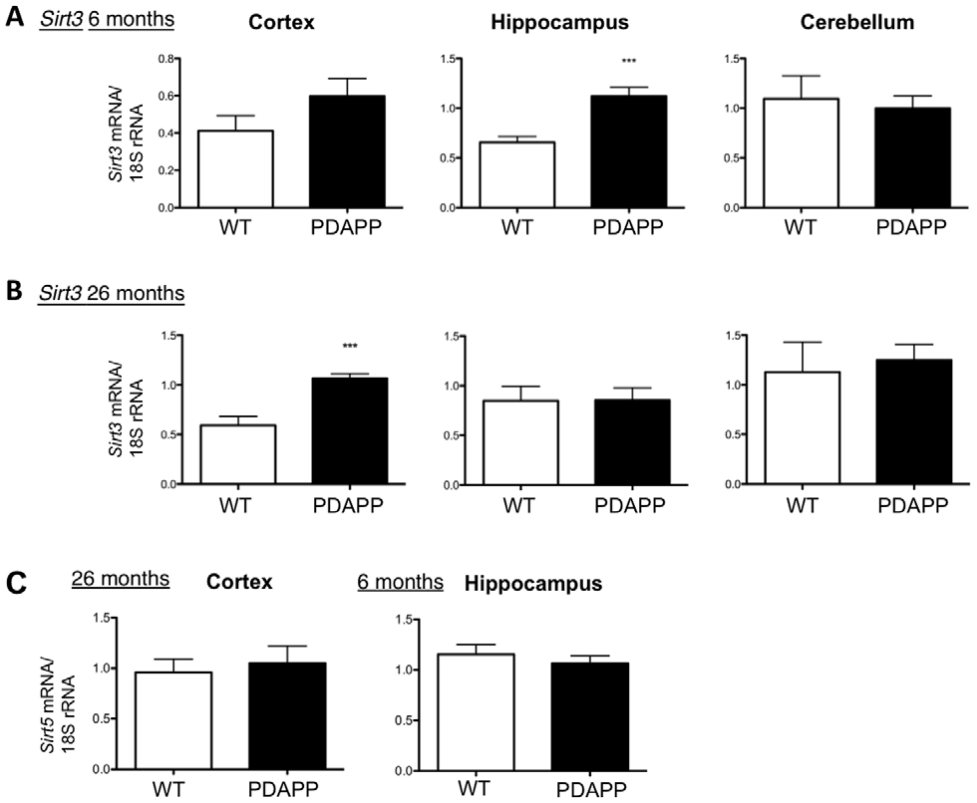
Sirt3 mRNA is upregulated in a specific spatio-temporal pattern in a mouse model of AD. **A.** *Sirt3* mRNA expression is significantly upregulated in hippocampus samples of 6 months-old PDAPP mice (n = 9−10, ***P<0.001) with no alteration in cortex (n = 10) and cerebellum (n = 10). *Sirt3* mRNA was measured and normalized to 18S rRNA using TaqMan multiplex QPCR. **B.** *Sirt3* mRNA expression is significantly upregulated in cortex samples of 26 months-old PDAPP mice (n = 6−7, ***P<0.001) with no alteration in hippocampus (n = 8) and cerebellum (n = 6−7). **C.** *Sirt5* mRNA is unaltered in 6 months-old hippocampus and 26 months-old cortex PDAPP samples.

As mutant APP and Aβ interact with mitochondrial proteins and increase ROS, we investigated whether *Sirt3* mRNA expression may be affected in this pathological background. We used a transgenic PDAPP mouse model, which over-expresses human APP carrying the V717F mutation [20]. These mice progressively develop many of the pathological hallmarks of AD, although they do not suffer significant neuronal loss nor demonstrate neurofibrillary tangle pathology [21]. To investigate *Sirt3* expression during disease progression, and in different CNS areas, we studied *Sirt3* mRNA in cortex, hippocampus and cerebellum of 6- and 26-month-old wild-type (WT) and PDAPP mice. Compared with WT littermates *Sirt3* mRNA was significantly upregulated in the PDAPP hippocampus at 6 months, while neither cortex nor cerebellum showed significant dysregulation at this stage (Fig. 3A). At 26 months, *Sirt3* mRNA was significantly increased in PDAPP cortex, while neither hippocampus nor cerebellum showed significant alterations (Fig. 3B). *Sirt3* mRNA expression thus mirrored the previously demonstrated spatiotemporal pattern of Aβ-deposition in this strain of PDAPP transgenic mice, occurring first in hippocampus, later in cortex and in very low amounts at any age in cerebellum [22]. *Sirt5* mRNA expression remained unchanged in CNS areas and at times where significant *Sirt3* mRNA changes were observed, suggesting that Aβ effects are specific for Sirt3 (Fig. 3C).

### Sirt3 Expression is Upregulated in Human Alzheimer’s Disease (AD)

**Figure 4:**
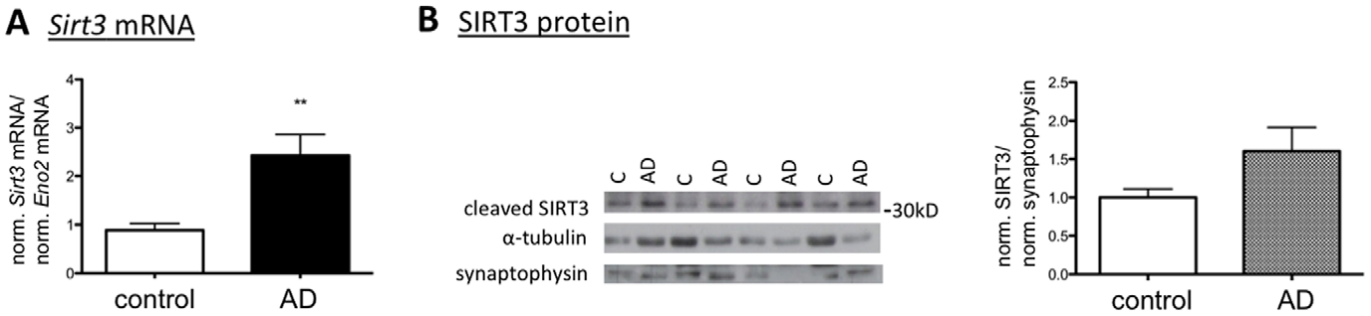
Sirt3 is upregulated in human Alzheimer’s disease (AD). **A.** *Sirt3* mRNA expression is significantly increased in AD temporal cortex samples compared to matched controls (n = 14, **P<0.01). Protein and RNA were extracted from neuropathologically confirmed sporadic AD and matched control temporal cortex dissected samples. *Sirt3* and *Eno2* mRNA were measured and normalized to 18S rRNA using TaqMan multiplex QPCR. **B.** Cleaved (active) SIRT3 protein is increased in AD temporal cortex samples (n = 14−15). Protein levels were determined by Western Blot and normalized to α-tubulin and synaptophysin.

As neurodegeneration in AD is associated with significant increases in neuronal ROS production [2], we investigated whether the expression of *Sirt3* might be altered in this disease in humans. We studied brain tissue from cases of neuropathologically confirmed sporadic AD and matched controls (Table S1 and Fig. S4), focusing on the temporal cortex as a brain region heavily affected in AD. Since AD is associated with significant neuronal cell loss, we calibrated *Sirt3* mRNA expression against expression of neuron-specific enolase (*Eno2*) [23]. *Sirt3* mRNA was significantly increased in the AD group (Fig. 4A). The level of cleaved (active) SIRT3 protein was also increased in temporal cortex from AD patients (Fig. 4B).

## Discussion

Collectively our *in vitro*, AD mouse model and human AD post mortem tissue data suggest a neuroprotective role for SIRT3 in CNS mechanisms dealing with mitochondrial stress, including during AD progression. Presumably this role for SIRT3 extends to other neurodegenerative diseases where mitochondrial oxidative stress is a key component of neuronal decline, including Parkinson’s disease and multiple sclerosis.

### CNS Sirt3 Localization

Although our finding of SIRT3 expression throughout the CNS in both neuronal and glial cell types might suggest ubiquitous importance for this protein, a CNS-specific phenotype for SIRT3-deficient mice has not yet been reported. Indeed, cochlear neurons in SIRT3-deficient mice appear normal, until challenged by a stressor such as calorie restriction, following which a failure to increase *Sirt3* expression leads to cell death [9]. These data suggest that CNS SIRT3 may be dispensable, until its upregulation is needed to protect neurons from oxidative stress. Our demonstration of *Sirt3* mRNA upregulation in response to ETC-mediated ROS induction and subsequent Sirt3-mediated increase in neuronal longevity supports this concept. Interestingly, Kim *et al.* recently described an increase in mitochondrial SIRT3 localization in primary cortical neurons in response to NMDA-mediated excitotoxicity [24], suggesting an important role for *Sirt3* mRNA splicing. However, our data show that long- or short-form-specific *Sirt3* splicing is not involved in the cellular response to mitochondrial stress. Nevertheless, we subsequently demonstrate that over-expression of long-form SIRT3 alone is neuroprotective; a physiological role for short-form SIRT3 remains to be established.

### Sirt3 Upregulation and Increase of Neuronal Lifespan in Mitochondrial Oxidative Stress

Pharmacological interference with the ETC to cause mitochondrial oxidative stress and subsequent scavenging of mitochondrial ROS modulate *Sirt3,* but not *Sirt5* mRNA expression, demonstrating that mitochondrial ROS per se has acute effects specifically on *Sirt3* mRNA expression levels. Whilst it is known that SIRT3 plays a role in regulating ROS levels, we demonstrate responsiveness of CNS *Sirt3* mRNA expression to mitochondrial ROS, suggesting SIRT3 regulation by the CNS mechanisms sensing mitochondrial health. Furthermore, we demonstrate that an increase specifically in long-form *Sirt3* results in significant extension of neuronal lifespan in the face of mitochondrial oxidative stress, although this is presumably just one of many mechanisms by which neurons respond to this pathology. Interestingly, SIRT3 was shown to be a prosurvival factor in NMDA-mediated excitotoxic injury *in vitro* [23].

### Sirt3 Upregulation in AD

Our data from PDAPP mice and human post-mortem AD samples demonstrate that *Sirt3* is upregulated in association with Aβ-accumulation. This constitutes the first demonstration of Sirt3 involvement in AD neurodegenerative disease. As AD is associated with significant increases in neuronal ROS production [2] and our *in vitro* data shows that *Sirt3* mRNA expression is regulated by mitochondrial ROS levels, it seems likely that *Sirt3* upregulation in AD may be a consequence of Aβ-related oxidative stress. Given that *Sirt3* upregulation subsequently increases neuronal lifespan, it is tempting to speculate that upregulation of SIRT3 in response to Aβ-induced oxidative stress might prolong neuronal function by decreasing ROS, maintaining ATP-levels and sustaining synaptic activity, but this will need further investigation. SIRT3 has indeed been demonstrated to reduce ROS and maintain ATP levels in other peripheral tissues [7].

In the PDAPP mouse, *Sirt3* mRNA upregulation mirrored spatiotemporal Aβ deposition. In PDAPP mice, Aβ levels rise first and ultimately to a much higher degree in the hippocampus, in comparison to cortex, while the cerebellum remains unaffected [22]. Similarly, *Sirt3* mRNA is upregulated in hippocampus at 6 months, while cortical *Sirt3* upregulation followed at a later stage (cerebellar *Sirt3* remained unaffected). Hippocampal *Sirt3* mRNA levels in PDAPP mice declined to wild-type levels at 26 months, perhaps reflecting failure of the *Sirt3* response at this late stage of the disease.

A clear difference between the PDAPP mouse model and human AD is the lack of neuronal loss in the mouse model [21]. Up-regulation of *Sirt3* mRNA in human AD postmortem tissue thus stems from *remaining* cells, perhaps in a bid to increase neuronal longevity, although this will need further clarification.

In addition, due to inevitable inter-individual variations in gene expression, as well as potentially variable tissue quality, human AD postmortem studies will need to be expanded to include a larger cohort of control and AD samples.

### Sirt3 vs. Sirt5

*In vitro* manipulation of ROS suggests, that CNS *Sirt3*, but not *Sirt5* expression is regulated in response to mitochondrial oxidative stress and the *in vivo* mechanisms leading to *Sirt3* induction during AD disease progression are also specific to Sirt3. Out data thus suggest that SIRT3 regulation during oxidative stress and Aβ-accumulation does not simply stem from increased mitochondrial biogenesis or global mitochondrial protein upregulation, but is a specific mechanism responding to increased ROS.

Collectively our data suggest an intriguing role for SIRT3 in the CNS processes attempting to maintain mitochondrial and ultimately neuronal health in the face of AD-induced mitochondrial stress. Further studies will need to investigate SIRT3’s neuroprotective role in AD-mediated neurodegeneration.

## Materials and Methods

Detailed methods can be found in the Supporting Information (Text S1).

### Primary Hippocampal Cell Culture

Isolation of primary rat hippocampal cultures was carried out as described previously [25]. Cultures were transfected 3 days post-isolation.

### Transfection and Immunofluorescence

Long-form and short-form SIRT3 were amplified from mouse brain cDNA and cloned in-frame into pEGFP N1 expression vector (Clontech Laboratories). HeLa and HEK293T cells and primary hippocampal cultures were transfected using FuGENE 6 (Roche Diagnostics GmBH, Mannheim, Germany) and Lipofectamine 2000 (Invitrogen Life Technologies) transfection reagents respectively, according to manufacturers’ instructions. Primary hippocampal cultures were fixed with 10% formalin and labeled immunofluorescently as previously described [25].

### cDNA Generation and Real-Time PCR

RNA was extracted from human tissue as described previously [23]. Human and mouse brain tissue was homogenized in TRIzol reagent (Invitrogen Life Technologies) and reverse transcribed according to manufacturer’s instructions. Multiplex Real-Time PCR was performed using TaqMan Assay-on-demand probes (Applied Biosystems, Foster City, CA).

### In vitro Antimycin A and NAC Treatment

6-day-old primary hippocampal cultures were loaded with MitoSOX (0.5 µM, Invitrogen Life technologies) according to manufacturers instructions. Cultures were pretreated with NAC (100 µM, Sigma) overnight prior to antimycin A (250 nM, Sigma) treatment in neurobasal medium and images acquired at 0 h and 12 h post-treatment. Fluorescence intensity was quantitated in ≥50 neurons using ImageJ.

### Lenti-mSIRT3iGFP Generation

Long-form mouse SIRT3 cDNA was cloned into pIRES2-EGFP (Clontech) upstream of the IRES-GFP sequences. The mSIRT3-IRES-GFP construct was then inserted into a lenti-synapsin plasmid (lenti-mSIRT3iGFP) and lentivirus generated and concentrated as described previously [25].

### Neuronal Death Analysis

Primary hippocampal cultures expressing lenti-mSIRT3iGFP or lenti-GFP were treated with AA (250 nM). Time-lapse microscopy was used to acquire images of the cultures at 5 min intervals and fluorescent neuronal death recorded by morphological changes including cell rounding.

### Mice

Mice were maintained on a 12-hour light/dark cycle with free access to water and mouse chow (2016 Teklad Global 16% Protein Rodent Diet, Harlan, UK). Studies were performed in accordance with the UK Animals (Scientific Procedures) Act and with approval of the University of Bristol Ethical Review Group. PDAPP mice have been described previously [20] and wild-type littermates were used as controls.

### Human Brain Tissue

Brain tissue was obtained from the Human Tissue Authority-licensed South West Dementia Brain Bank, University of Bristol, with North Somerset and South Bristol Research Ethics Committee approval. Frozen tissue was dissected from the midfrontal and temporal neocortex (Brodmann areas 6 and 22).

### Immunoblotting

Western Blot analysis was performed as previously described [25].

### Statistical Analysis

Data were analysed in GraphPad Prism using Student’s t-test and one-way ANOVA with Tukey’s Post hoc test.

## Supporting Information

Figure S1 **Mouse CNS SIRT3 expression.** SIRT3 is expressed in most areas of the mouse CNS. **A** Coronal mouse brain sections with (right panel) and without (left panel) anti-mouse SIRT3 anti-body immunohistochemistry. Magnified areas show cortical and hippocampal SIRT3 expression. Scale bar 500 µm **B** Co-localization with NeuN shows SIRT3 expression in neuronal and non-neuronal cells**.** Scale bar 50 µm.
(TIF)

Figure S2 **Expression of Sirt3 splice variants in response to AA treatment in mouse primary hippocampal neurons.** TaqMan qPCR probes were designed to specifically measure expression of long-form or short-form Sirt3. PC12 cells were transfected with plasmids expressing either long-form or short-form SIRT3 to test the specificity of the probes. Sirt3 expression was measured using probes designed to bind to all Sirt3 splice forms (**A**), only long-form Sirt3 (**B**), or only short-form Sirt3 (**C**) n = 3. **D/E/F** Mouse primary hippocampal neurons were treated with AA (250 nM) for 12 h. *Sirt3* mRNA expression was measured using the probe to measure either total Sirt3 (**D**), long-form Sirt3 (**E**) or short-form Sirt3 (**F**). **G** Ratio of long-form Sirt3/short-form Sirt3. n = 6. Student’s t-test: **p<0.01, *p<0.05.
(TIF)

Figure S3 **Lentiviral mouse Sirt3 over-expression.** Neuronal-specific mouse *Sirt3* over-expression increases neuronal longevity in the face of ROS augmentation. **A** Phase (left panel) and fluorescent (right panel) images of primary hippocampal neurons expressing the lenti-mSIRT3iGFP construct. **B** and **C** *Sirt3* over-expression was measured in rat primary hippocampal cultures (n = 4) that had been transduced with either lenti-GFP, lenti-mSIRT3iGFP or untransduced (control). mRNA expression was measured by qPCR relative to 18S rRNA using a TaqMan probe specific for mouse *Sirt3* (exogenous, **B**) or rat *Sirt3* (endogenous, **C**). **D** Representative survival curve of neurons expressing either GFP or mSIRT3iGFP lentivirus and treated with AA (250 nM). **E** Mean time of death of neurons from D. (n = 34–48, ***P>0.0001)
(TIF)

Figure S4 **Transcript stability and PM delay.** No correlation between 18S rRNA or *Sirt3* and *Eno2* mRNA expression with respect to PM delay in human control and AD brain samples.
(TIF)

Table S1 **Details of human cases studied.**
(DOCX)

Text S1 **Extended materials and methods.**
(DOCX)
