# Supplementary figures and images for: CNS SIRT3 Expression Is Altered by Reactive Oxygen Species and in Alzheimer’s Disease
Source: PLoS One. 2012 Nov 6;7(11):e48225. doi: 10.1371/journal.pone.0048225 (PMC3491018; doi:10.1371/journal.pone.0048225)

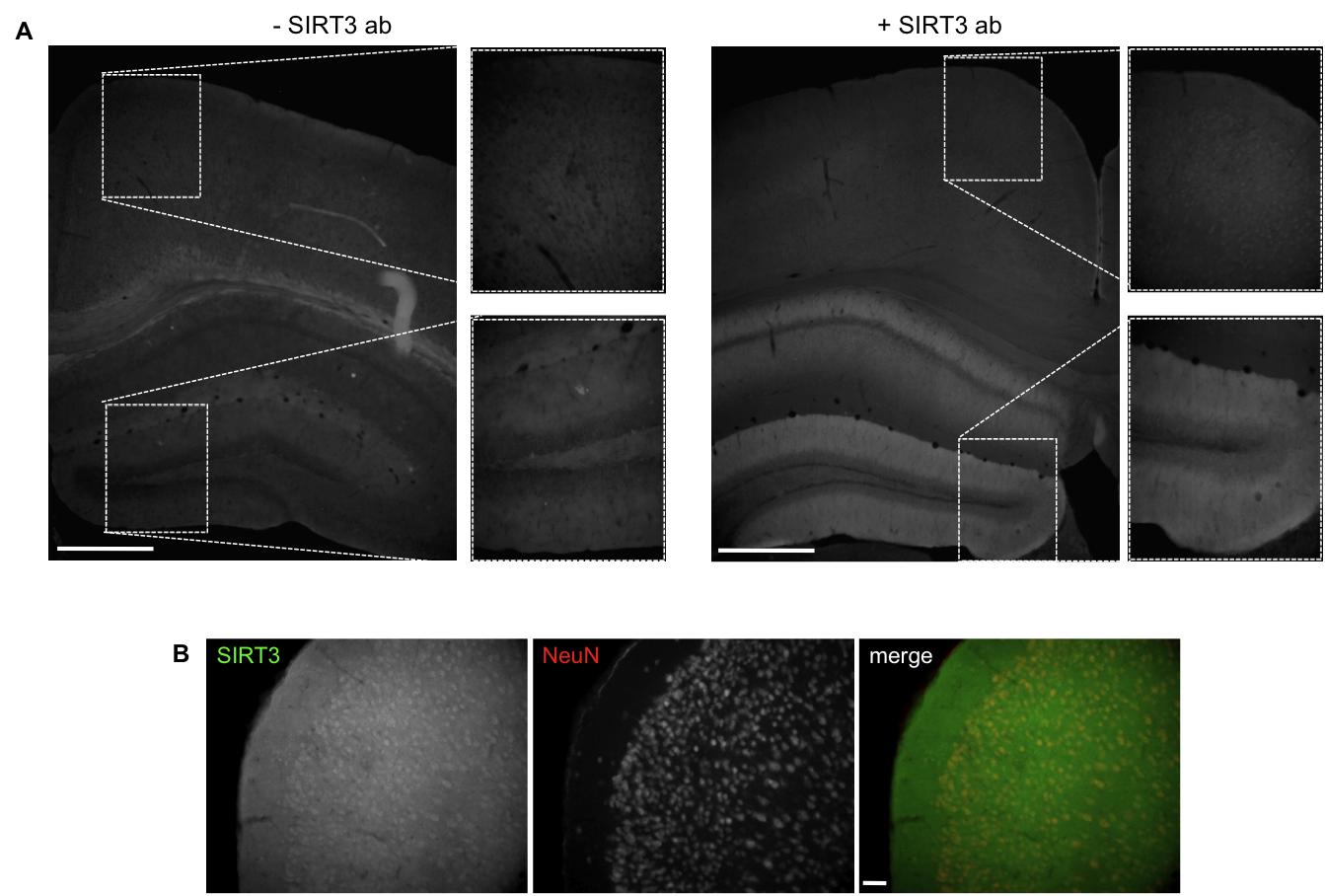

Supplement: Figure S1 — Mouse CNS SIRT3 expression. SIRT3 is expressed in most areas of the mouse CNS. A Coronal mouse brain sections with (right panel) and without (left panel) anti-mouse SIRT3 anti-body immunohistochemistry. Magnified areas show cortical and hippocampal SIRT3 expression. Scale bar 500 µm B Co-localization with NeuN shows SIRT3 expression in neuronal and non-neuronal cells. Scale bar 50 µm. (TIF) [file pone.0048225.s001.tif]

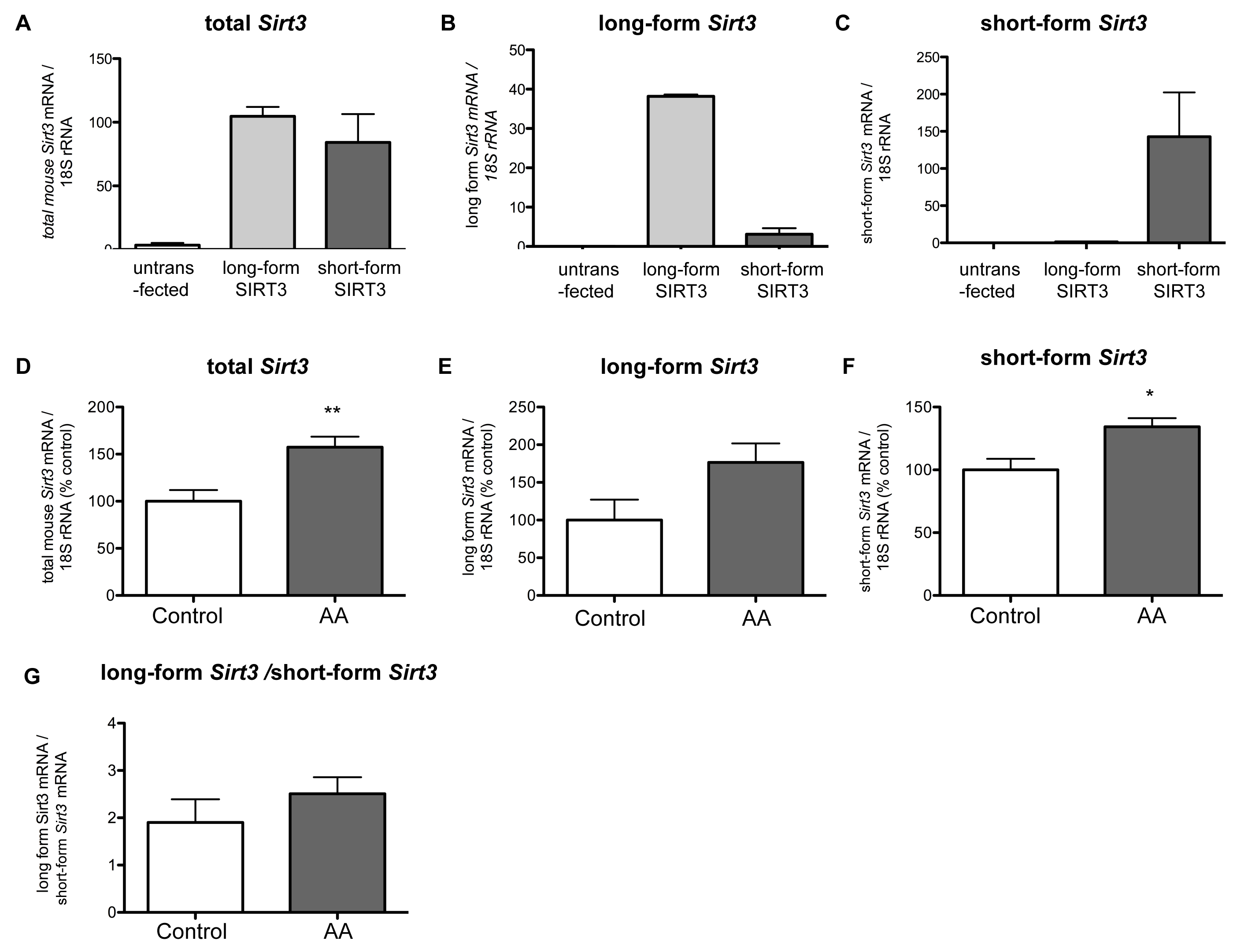

Supplement: Figure S2 — Expression of Sirt3 splice variants in response to AA treatment in mouse primary hippocampal neurons. TaqMan qPCR probes were designed to specifically measure expression of long-form or short-form Sirt3. PC12 cells were transfected with plasmids expressing either long-form or short-form SIRT3 to test the specificity of the probes. Sirt3 expression was measured using probes designed to bind to all Sirt3 splice forms (A), only long-form Sirt3 (B), or only short-form Sirt3 (C) n = 3. D/E/F Mouse primary hippocampal neurons were treated with AA (250 nM) for 12 h. Sirt3 mRNA expression was measured using the probe to measure either total Sirt3 (D), long-form Sirt3 (E) or short-form Sirt3 (F). G Ratio of long-form Sirt3/short-form Sirt3. n = 6. Student’s t-test: **p<0.01, *p<0.05. (TIF) [file pone.0048225.s002.tif]

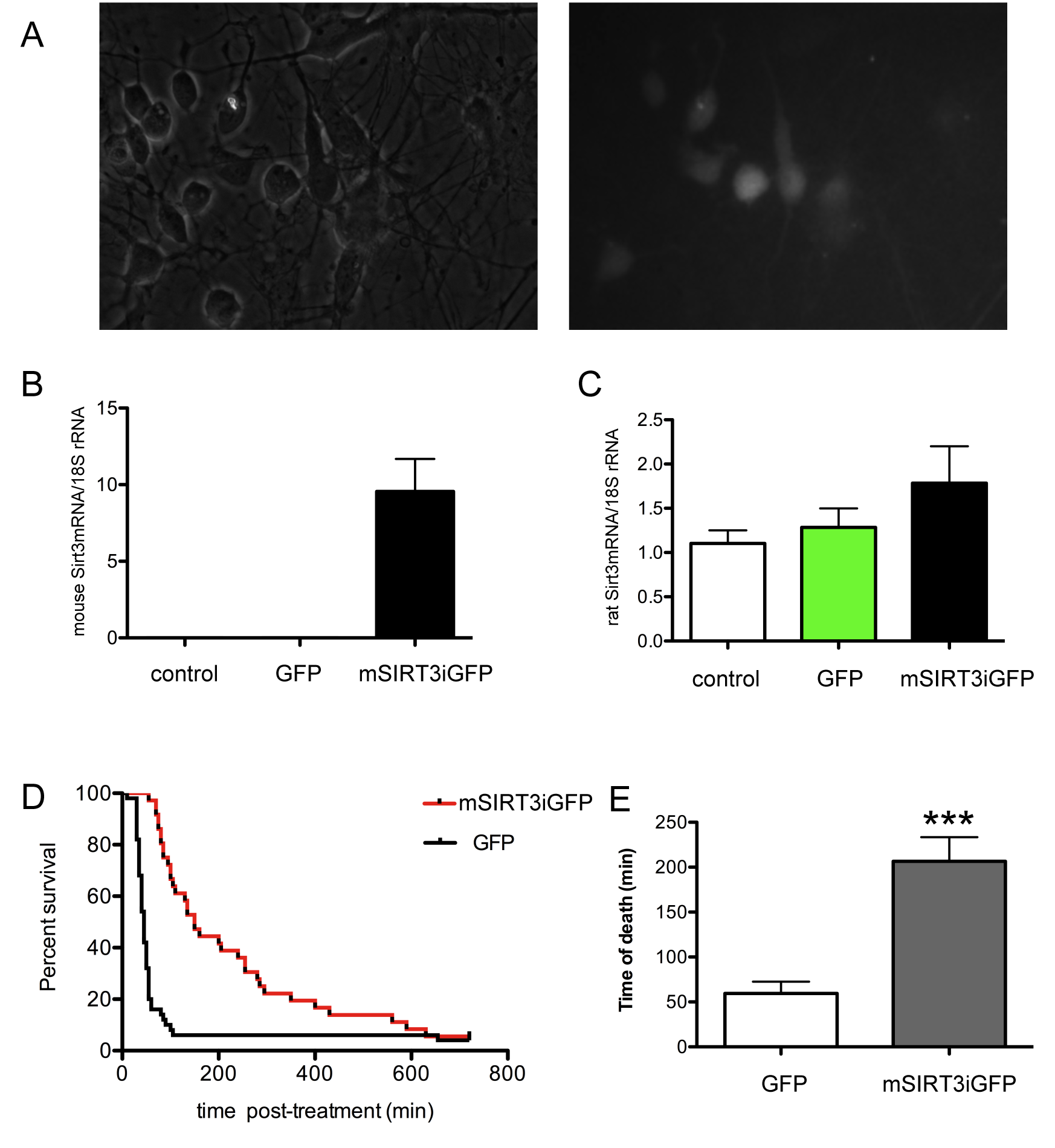

Supplement: Figure S3 — Lentiviral mouse Sirt3 over-expression. Neuronal-specific mouse Sirt3 over-expression increases neuronal longevity in the face of ROS augmentation. A Phase (left panel) and fluorescent (right panel) images of primary hippocampal neurons expressing the lenti-mSIRT3iGFP construct. B and C Sirt3 over-expression was measured in rat primary hippocampal cultures (n = 4) that had been transduced with either lenti-GFP, lenti-mSIRT3iGFP or untransduced (control). mRNA expression was measured by qPCR relative to 18S rRNA using a TaqMan probe specific for mouse Sirt3 (exogenous, B) or rat Sirt3 (endogenous, C). D Representative survival curve of neurons expressing either GFP or mSIRT3iGFP lentivirus and treated with AA (250 nM). E Mean time of death of neurons from D. (n = 34–48, ***P>0.0001) (TIF) [file pone.0048225.s003.tif]

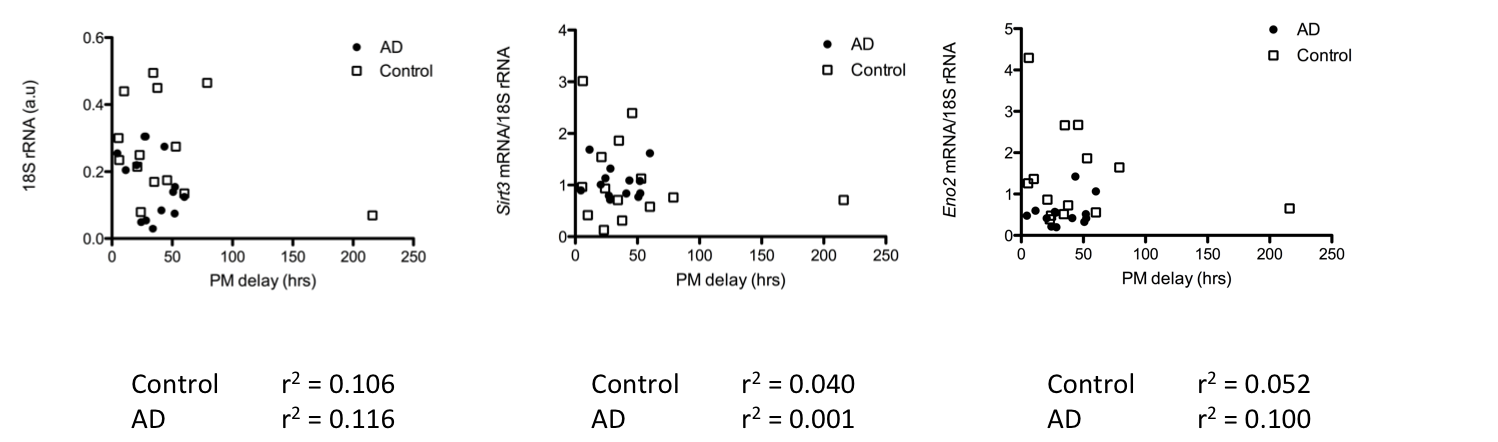

Supplement: Figure S4 — Transcript stability and PM delay. No correlation between 18S rRNA or Sirt3 and Eno2 mRNA expression with respect to PM delay in human control and AD brain samples. (TIF) [file pone.0048225.s004.tif]
