# Supplementary material for: CNS SIRT3 Expression Is Altered by Reactive Oxygen Species and in Alzheimer’s Disease
Source: PLoS One. 2012 Nov 6;7(11):e48225. doi: 10.1371/journal.pone.0048225 (PMC3491018; doi:10.1371/journal.pone.0048225)
Supplement: Table S1 — Details of human cases studied. (DOCX) [file pone.0048225.s005.docx]

|  | **Age** | **Sex** | **Braak stage** | **PM delay** |
| --- | --- | --- | --- | --- |
| **Control** |  |  |  |  |
| average±SEM | 82.3±3.1 | 9M 6F | median=II | 49.8±13.6 |
| Range | 73-94 |  | 0-III | 5.5-216 |
| **AD** |  |  |  |  |
| average±SEM | 82.5±2.3 | 7M 8F | median=IV | 34.1±4.4 |
| Range | 65-97 |  | III-VI | 4.5-52.3 |

Table S1: Details of human cases studied.
