# Supplementary material for: CNS SIRT3 Expression Is Altered by Reactive Oxygen Species and in Alzheimer’s Disease
Source: PLoS One. 2012 Nov 6;7(11):e48225. doi: 10.1371/journal.pone.0048225 (PMC3491018; doi:10.1371/journal.pone.0048225)
Supplement: Text S1 — Extended materials and methods. (DOCX) [file pone.0048225.s006.docx]

Extended Materials and Methods:

*Transfection and immunofluorescence*. To generate long- and short-form SIRT3-eGFP fusion construct, long- and short-form SIRT3 was amplified from mouse brain cDNA and cloned in-frame into pEGFP N1 expression vector (Clontech Laboratories). HeLa and HEK293T cells and primary hippocampal cultures were transfected using FuGENE 6 (Roche Diagnostics GmBH, Mannheim, Germany) and Lipofectamine 2000 (Invitrogen Life Technologies) transfection reagents respectively, according to manufacturers’ instructions. Primary hippocampal cultures fixed with 10% formalin were labeled immunofluorescently with anti-GFP (1:2000, Invitrogen Life Technologies), anti-HSP60 (1:1000, Sigma) anti-NeuN (1:200, Abcam) and anti-SIRT3 (1:500, a gift from E. Verdin, UCSF, USA) as previously described [[24](#_ENREF_24)]. Imaging was on a **Leica AOBS SP2 confocal imaging system.**

*cDNA Generation and Real-Time PCR.* RNA was extracted from human tissue as described previously[[22](#_ENREF_22)]. Human brain and mouse tissue was homogenized in TRIzol reagent (Invitrogen Life Technologies) according to manufacturer’s instructions. The RNA was DNase treated (Ambion Inc, Austin, TX) and cDNA generated with RevertAid First Strand cDNA Synthesis Kit (Fermentas, UK). Multiplex Real-Time PCR was performed on a Stratagene Mx3000P system (Stratagene, Cedar Creek, TX) using 18S rRNA (Applied Biosystems, Foster City, CA) and TaqMan Assay-on-demand probes for *Sirt3*, *Eno2* and *Sirt5* (*Sirt3*: Rn01501410_m1, Hs00202030_m1 and Mm00452129_m1, *Eno2*: Hs00157360_m1, *Sirt5*: Rn01450559_m1 and Mm01351576_m1; Applied Biosystems, Foster City, CA). To analyse the expression of long-form Sirt3 and short-form Sirt3 specifically, TaqMan probes were designed using the Custom TaqMan Assay Design Tool (Applied Biosystems). The amplicon sequence began just upstream of exon 2, either lacking (for long form Sirt3) or including (for short-form Sirt3) the sequence TGTTACAG – the run of 8bp by which the splice variants differ.

*In vitro antimycin A and NAC treatment*. To measure mitochondrial ROS production, 6-day-old primary hippocampal cultures were loaded with MitoSOX (0.5μM, Invitrogen Life technologies) according to manufacturers instructions. Cultures were pretreated with NAC (100μM, Sigma) overnight prior to antimycin A (250nM, Sigma) treatment in neurobasal medium and images acquired at 0h and 12h post-treatment. Wide-field epifluorescence images were obtained using an Olympus IX-71 inverted microscope (60× Uplan Fluorite objective 0.65-1.25 NA, at maximum aperture) fitted with a CoolSNAP HQ CCD camera (Photometrics, Tucson, AZ) driven by MetaMorph software (Molecular Devices, Sunnyvale, CA). Fluorescence intensity was quantitated in ≥50 neurons using ImageJ.

*Neuronal death analysis*. Primary hippocampal cultures expressing lenti-mSIRT3iGFP or lenti-GFP were treated with AA (250nM). Time-lapse microscopy was used to acquire images of the cultures at 5 min intervals and fluorescent neuronal death recorded by morphological changes including cell rounding. Neuronal death was assessed in 3 separate experiments with analysis of >30 fluorescent neurons/experiment. Data was normalized to control for each experiment. Fig. 2d shows pooled data, Fig.S2d,e shows representative experiment.

*Human brain tissue.* Brain tissue was obtained from the Human Tissue Authority-licensed South West Dementia Brain Bank, University of Bristol, with local Research Ethics Committee approval. Frozen tissue was dissected from the midfrontal and temporal neocortex (Brodmann areas 6 and 22) from 15 AD cases and 15 controls in which transcript stability could be demonstrated with respect to post mortem (PM) delay (See Fig. S4). The AD cases had a diagnosis according to the Consortium to Establish a Registry for Alzheimer’s disease of 'deﬁnite AD' [[25](#_ENREF_25)] and a Braak tangle stage of IV–VI [[26](#_ENREF_26)]. The controls had no history of dementia, few or no neuritic plaques, a Braak tangle stage of III or less, and no other neuropathological abnormalities (Table S1).

|  | **Age** | **Sex** | **Braak stage** | **PM delay** |
| --- | --- | --- | --- | --- |
| **Control** |  |  |  |  |
| average±SEM | 82.3±3.1 | 9M 6F | median=II | 49.8±13.6 |
| Range | 73-94 |  | 0-III | 5.5-216 |
| **AD** |  |  |  |  |
| average±SEM | 82.5±2.3 | 7M 8F | median=IV | 34.1±4.4 |
| Range | 65-97 |  | III-VI | 4.5-52.3 |

Table S1: Details of human cases studied.

*Immunoblotting.* Human brain tissue was prepared for immunoblotting by homogenization in lysis buffer (100mM NaCl, 10mM Tris pH7.6, 1ng/ml aprotinin, 1nM PMSF, 1% SDS). Protein samples were separated by SDS-PAGE on 12% Tris-HCl gels and membranes were blotted with anti-SIRT3 (1:500, Abgent), anti-tubulin (1:10000, Sigma) and anti-synaptophysin (1:1000, Abcam).

Supplemental References:

25. Mirra SS, Heyman A, McKeel D, Sumi SM, Crain BJ, et al. (1991) The Consortium to Establish a Registry for Alzheimer's Disease (CERAD). Part II. Standardization of the neuropathologic assessment of Alzheimer's disease. Neurology 41: 479-486.

26. Braak H, Braak E, Bohl J (1993) Staging of Alzheimer-related cortical destruction. Eur Neurol 33: 403-408.
